# Inverse Identification of Constitutive Model for GH4198 Based on Genetic–Particle Swarm Algorithm

**DOI:** 10.3390/ma17174274

**Published:** 2024-08-29

**Authors:** Qichao Jin, Jun Li, Fulin Li, Rui Fu, Hongyu Yu, Lei Guo

**Affiliations:** 1 Key Laboratory of Road Construction Technology and Equipment of MOE, Chang’an University, Xi’an 710064, China; junli070@126.com (J.L.); yhysx1999@126.com (H.Y.); lguo@chd.edu.cn (L.G.); 2 AECC Xi’an Aero-Engine Ltd., Xi’an 710021, China; 3 GaoNa Aero Material Co., Ltd., Beijing 100081, China; lifulin1016@sina.com (F.L.); furui208@sina.com (R.F.)

**Keywords:** cast and wrought alloy GH4198, constitutive model, inverse identification, orthogonal cutting, finite-element model, genetic particle swarm optimization

## Abstract

A precise Johnson-Cook (J–C) constitutive model is the foundation for precise calculation of finite-element simulation. In order to obtain the J–C constitutive model accurately for a new cast and forged alloy GH4198, an inverse identification of J–C constitutive model was proposed based on a genetic–particle swarm algorithm. Firstly, a quasi-static tensile test at different strain rates was conducted to determine the initial yield strength *A*, strain hardening coefficient *B*, and work hardening exponent *n* for the material’s J–C model. Secondly, a new method for orthogonal cutting model was constructed based on the unequal division shear theory and considering the influence of tool edge radius. In order to obtain the strain-rate strengthening coefficient *C* and thermal softening coefficient *m*, an orthogonal cutting experiment was conducted. Finally, in order to validate the precision of the constitutive model, an orthogonal cutting thermo-mechanical coupling simulation model was established. Meanwhile, the sensitivity of J–C constitutive model parameters on simulation results was analyzed. The results indicate that the parameter *m* significantly affects chip morphology, and that the parameter *C* has a notable impact on the cutting force. This study addressed the issue of missing constitutive parameters for GH4198 and provided a theoretical reference for the optimization and identification of constitutive models for other aerospace materials.

## 1. Introduction

The new cast and forged turbine disk alloy GH4198 shows promising application prospects in aerospace and aviation engines. GH4198 high-temperature alloy, as a crucial material for high-performance aerospace engine discs and other hot-end components, stands out as one of the best turbine disk alloys for operating conditions approaching 800 °C [1,2,3]. GH4198 alloy is known for its challenging machinability. It encounters issues such as high cutting forces, elevated cutting temperatures, and difficulties in controlling the quality of machined surfaces. Constitutive models are used to describe the plastic deformation of materials during cutting processes [4]. Recently, there has been no research on the constitutive model for GH4198 alloy, hence hindering the construction of an accurate finite-element model for analyzing cutting processes. Consequently, the inability to develop rational cutting processes to control machining deformation and precision limits its widespread application in various critical fields. Developing an accurate constitutive model for GH4198 is essential to provide a theoretical basis for studying its machining performance. Therefore, developing an accurate constitutive model to characterize metal flow behavior is crucial for optimizing cutting processes and enhancing the surface quality of workpieces.

Constitutive models are crucial for describing thermomechanical coupling effects, which can be categorized into empirical and physical types [5,6]. The reliability of the finite-element model is closely tied to the constitutive model of materials. The J–C constitutive model considers the effects comprehensively of material strain hardening, strain-rate strengthening, and temperature softening [7], as depicted in Equation (1). This model accurately describes the plastic deformation of metals under conditions of large deformations, high strain rates, and elevated temperatures [8,9,10]. It is widely utilized to study the mechanical behavior of materials in machining processes, and is among the most frequently utilized material models in finite-element simulations of machining [9].

(1) $$\tau_{\mathrm{AB}}=\frac{1}{\sqrt{3}}\left[A+B{\varepsilon_{\mathrm{AB}}}^{n}\right]\left[1+C\ln\left(\frac{{\dot{\varepsilon }}_{\mathrm{AB}}}{{\dot{\varepsilon }}_{0}}\right)\right]\left[1-{\left(\frac{T_{\mathrm{AB}}-T_{r}}{T_{w}-T_{r}}\right)}^{m}\right]$$

In the equation, *τ*_AB_ represents the equivalent shear stress, $\varepsilon_{\mathrm{AB}}$ denotes the equivalent plastic strain, ${\dot{\varepsilon }}_{AB}$ stands for the equivalent strain rate, ${\dot{\varepsilon }}_{0}$ represents the equivalent reference strain rate, and *T*_AB_, *T_w_*, and *T*_m_ respectively denote the deformation temperature, workpiece material melting point, and ambient temperature. The physical meanings of constitutive parameters *A*, *B*, *C*, *m*, and *n* are the material’s initial yield strength, strain hardening coefficient, strain rate sensitivity coefficient, thermal softening coefficient, and work hardening exponent, respectively.

Recently, methods for obtaining a material J–C constitutive model have been divided into direct and indirect methods. Direct methods include the split Hopkinson pressure bar (SHPB) test and impact test. The indirect method integrates the slip line field theory model and orthogonal cutting test, employing optimization algorithms to identify the J–C constitutive model [11,12]. Direct methods generally refer to experimental approaches for directly obtaining the material J–C constitutive model. Grązka et al. [13] obtained the stress–strain curve for plastic hardening using the SHPB test, and derived the constitutive model through curve fitting. Tang [14] employed the downhill simplex method to optimize the J–C constitutive model and established a finite-element model based on the J–C model obtained from the indirect and direct method, confirming that the former exhibits higher accuracy in predicting cutting forces. Shen et al. [15] employed the SHPB test and parameter identification techniques to analyze the influence of the constitutive model on flow stress. This analysis revealed that the constitutive model obtained from the SHPB test inadequately characterizes the thermo-plastic deformation of materials during high-strain-rate cutting processes. The indirect method is to obtain the constitutive model indirectly by establishing theoretical and numerical models. Tounsi et al. [16] derived the J–C constitutive model based on orthogonal cutting theory and the least squares method. It was found that there is not a unique convergent solution for the inverse identification of the constitutive model based on indirect methods. Chen et al. [17], based on the Oxley unequal division shear model and orthogonal cutting test, employed the genetic algorithm to achieve inverse identification of the J–C constitutive model for GH4169 alloy. However, this method requires a predetermined range for the constitutive model to maintain algorithm convergence speed and identification accuracy. Wang et al. [18] conducted a tensile test and orthogonal cutting test, and employed a hybrid particle swarm algorithm to obtain the J–C constitutive model for ZM5 magnesium alloy. Li et al. [19] utilized the particle swarm algorithm for inverse identification to determine the J–C constitutive model for 316H stainless steel. Nguyen et al. [20] employed the theoretical model, considering the variation in chip thickness for each tooth during milling, and obtained the J–C constitutive model based on a genetic algorithm. Bäkera et al. [21] conducted an orthogonal cutting experiment, and employed the Levenberg–Marquardt optimization algorithm to obtain the J–C constitutive model. Tian et al. [22] employed the theoretical model, considering the effect of tool nose radius on cutting forces, combined with an optimization algorithm to obtain the J–C constitutive model. He et al. [23] obtained a TANH (Tangent Hyperbolic) constitutive model based on the particle swarm optimization algorithm, and established a connection between simulation observables and the constitutive model. Zou et al. [24] implemented automatic modeling and computation of a three-dimensional turning finite-element model using Python, and identified the J–C constitutive model based on a multi-island genetic algorithm. Zhou et al. [25] proposed a collaborative simulation-based identification method for the J–C constitutive model using genetic algorithms based on ABAQUS secondary development, and validated the effectiveness of the constitutive model by comparing residual stresses and chip characteristics between the cutting experiment and simulation. In summary, obtaining precise cutting simulation results for a constitutive model directly is challenging. Therefore, unequal division shear theory based on the indirect method is an effective approach for studying the GH4198 constitutive model.

In order to solve the problem of a missing constitutive model of GH4198 material, a novel inverse identification approach was proposed in this study. This paper conducted static tensile tests to determine parameters *A*, *B*, and *n*. Then, it employed the modified Oxley unequal division shear model and a genetic–particle swarm algorithm based on orthogonal cutting experimental data to identify parameters *C* and *m*. Finally, comparisons between the cutting experiment and simulation regarding cutting forces and chip geometrical features validated the effectiveness of the constitutive model. Meanwhile, this paper analyzed the impact of the strain-rate strengthening coefficient *C* and the thermal softening coefficient *m* on simulation observables.

## 2. Quasi-Static Tensile Test

**Figure 1:**
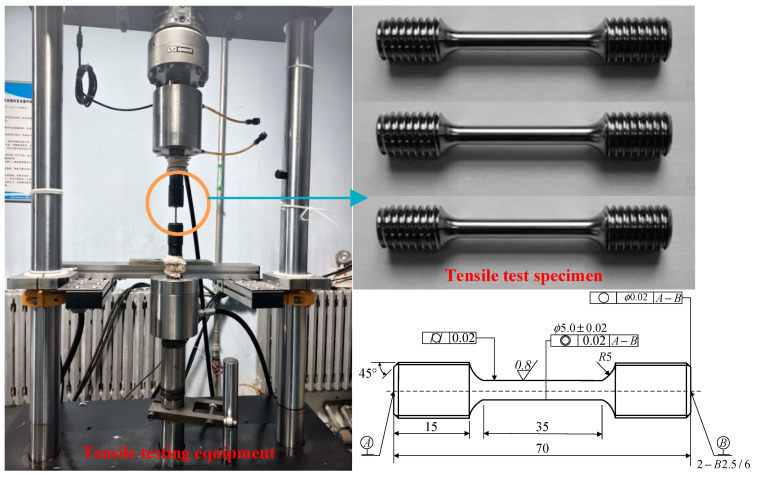
Tensile testing equipment and specimens.

GH4198 alloy is a nickel-based precipitation-strengthened deformation high-temperature alloy, with a γ′ phase volume fraction of approximately 53% [1], as shown in Table 1. The quasi-static tensile test was conducted on the INSTRON 8801 universal testing machine under three different reference strain rates,$\;{\dot{\varepsilon }}_{0}$: 0.01 s^−1^, 0.001 s^−1^, and 0.0002 s^−1^. GH4198 high-temperature alloy specimens were adopted, which underwent a series of processes including wire cutting to achieve specific dimensions, as shown in Figure 1. After completion of the tensile tests, relevant data measured by the equipment were recorded, as shown in Table 2.

**Figure 2:**
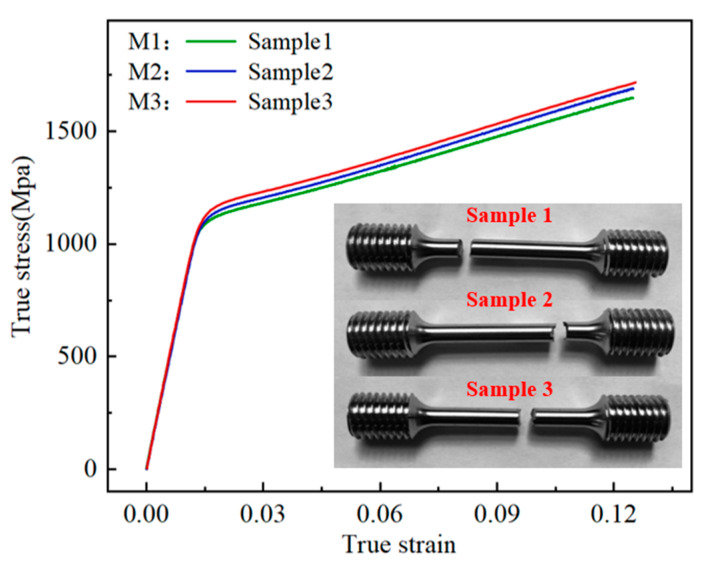
True stress–strain curves under different strain rates.

During the quasi-static stretching process and before the necking point formed, the area of the entire measurement section uniformly decreased. The true strain was calculated through measuring the relative displacement [26]. In this study, five sets of preliminary tensile tests were conducted at a strain rate of 0.01, showing good consistency with a maximum standard deviation of 0.016 in the true strain. The true stress–strain curves were computed based on the load–displacement data from the tensile tests. M1, M2, and M3 represented specimens respectively tested at different reference strain rates, as shown in Figure 2.

Quasi-static tensile testing was conducted at room temperature, where strain rate strengthening and thermal softening effects in the J–C constitutive model were neglected. Consequently, the J–C constitutive model could be simplified to:

(2) $$\sigma =A+B\varepsilon^{n}$$

To determine the strain hardening coefficient (*B*) and the work hardening exponent (*n*), Equation (1) was simplified as follows:

(3) ln(σ−A)=lnB+nlnε

The stress value at 0.2% plastic deformation was taken as the initial yield strength of the GH4198 high-temperature alloy based on the true stress–strain curves obtained from the quasi-static tensile testing at room temperature. The initial yield strength *A*, strain hardening coefficient *B*, and work hardening exponent *n* were determined for different reference strain rates based on MATLAB(2021b) fitting, as shown in Table 3. When the strain rate was low, decreasing the strain rate resulted in an increasing trend in the material’s initial yield strength and work hardening exponent, while the strain hardening coefficient showed a decreasing trend.

## 3. Orthogonal Cutting Experiment and Model

### 3.1. Orthogonal Cutting Experiment

**Figure 3:**
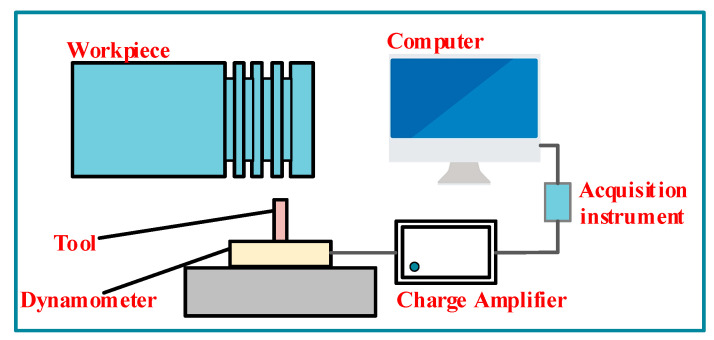
Principle diagram of orthogonal cutting test.

**Figure 4:**
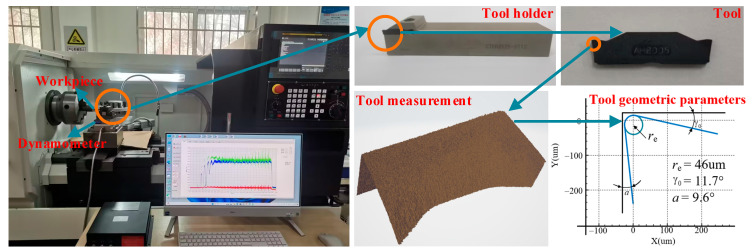
Orthogonal cutting equipment.

The principle of the orthogonal cutting test is shown in Figure 3. The experiment was carried out on a CNC lathe SK66Q, for which the equipment setup is shown in Figure 4. The test employed a dry cutting method with a cutting width of 2 mm. Cutting forces during the process were measured using an XR-YDCL-III89B triaxial piezoelectric force sensor (Shaanxi Xuanrui Electrical Automation Equipment Co., Ltd., Xi’an, China). The workpiece was a GH4198 high-temperature alloy rod with a diameter of 50 mm, and the tool was an AH8005 cemented carbide cutting tool. The tool geometry was measured using a Zoller profile scanner (Zoller Precision Testing Instruments Co., Ltd., Shanghai, China), revealing a rake angle $\gamma_{0}\;$ of 11.7°, clearance angle of 9.6°, and cutting-edge blunt round radius of 46 um. After cutting, the collected chips were embedded, polished, and then observed for geometric features using a 5XC-PC inverted metallographic microscope (Shanghai Optical, Shanghai, China). In order to ensure the validity of the data, six measurements were taken on the chip samples under each set of parameters to calculate the average chip thickness, saw-tooth degree, and shear angle.

### 3.2. Orthogonal Cutting Model

**Figure 5:**
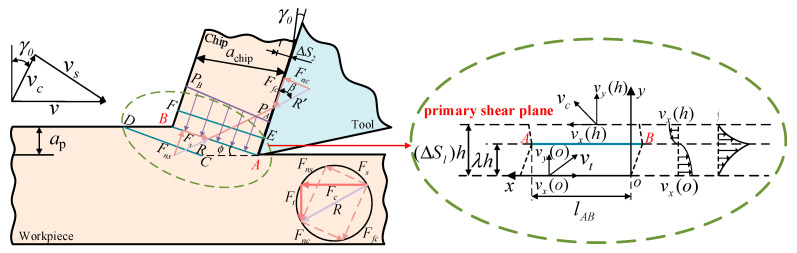
Schematic diagram of unequal shear zone model.

Unequal division shear theory is an advanced shear theory designed to more accurately describe the deformation behavior of materials under shear loads. This theory takes into account various irregularities present in real materials, positing that there may be differences in shear strain within the material. Recently, many studies have extended and developed cutting models that are more realistic for machining operations based on the Oxley parallel shear model [27]. In this paper, a stress, strain, strain rate, and temperature calculation model for the primary shear zone were constructed based on the unequal division shear model and the nonlinear shear strain rate distribution model proposed by Pang et al. [28]. The schematic diagram of the unequal division shear model is shown in Figure 5.

The expressions for the friction force (*F*_nc_) along the tool front face, normal force (*F*_fc_), and the shear force (*F*_s_) at shear plane AB, as well as the normal force (*F*_ns_), were calculated by Equation (4) based on the geometric relationships shown in Figure 5.

(4) $$\left\{ \begin{array}{l} F_{\mathrm{fc}}=F_{c}\sin\gamma_{0}+F_{t}\cos\gamma_{0}\\ F_{\mathrm{nc}}=F_{c}\cos\gamma_{0}-F_{t}\sin\gamma_{0}\\ F_{s}=F_{c}\cos\phi -F_{t}\sin\phi \\ F_{\mathrm{ns}}=F_{c}\sin\phi +F_{t}\cos\phi \end{array}\right.$$

The range of shear angle ϕ values is between 10° and 45°, and its final value was determined through iterative enumeration.

The formula describing the relationship among cutting speed (*v*), chip velocity (*v*_c_), and shear velocity (*v*_s_) was calculated by Equation (5) based on the geometric relationships of speed depicted in Figure 5.

(5) $$\left\{ \begin{array}{l} v_{c}=v\frac{\sin\phi }{\cos(\phi -\gamma_{0})}\\ v_{s}=v\frac{\cos\gamma_{0}}{\cos(\phi -\gamma_{0})} \end{array}\right.$$

The calculation formula for the friction angle (*β*) was derived from Equation (6).

(6) $$\beta =\arctan\left(\frac{F_{\mathrm{fc}}}{F_{\mathrm{nc}}}\right)$$

The formula for the average shear stress on shear plane AB was derived as follows:

(7) $$\tau_{\mathrm{AB}}=\frac{F_{s}\sin\phi }{a_{p}w}$$

It is assumed that the shear strain *τ* remained constant along the thickness *S*_1_ of the first deformation zone and that the strain rate at the primary shear plane AB reached its maximum value. Furthermore, the primary shear zone was considered as an adiabatic shear process, and variations in temperature gradient were negligible during the cutting process. Therefore, the static hydraulic pressure at points A and B could be derived by Equation (1):

(8) $$\left\{ \begin{array}{l} P_{A}=\tau_{\mathrm{AB}}\left[1+2\left(\frac{\pi }{4}-\phi \right)\right]\\ P_{B}=\tau_{\mathrm{AB}}\left[1+2\left(\frac{\pi }{4}-\phi \right)-\frac{C_{0}(q+1)}{\lambda l_{\mathrm{AB}}}n_{\mathrm{eq}}\right] \end{array}\right.$$

The constant *C*_0_ is the shear strain rate constant, defined as the ratio of the length *l*_AB_ of shear plane AB to the thickness *h* of the primary shear zone. The calculation formula for the shear zone thickness *h* was calculated by Equation (9):

(9) $$h=\frac{2a_{1}\tau_{\mathrm{AB}}}{(P_{A}-P_{B})\sin\phi }n_{\mathrm{eq}}$$

(10) $$n_{\mathrm{eq}}=\frac{nB\varepsilon_{\mathrm{AB}}^{n}}{A+B\varepsilon_{\mathrm{AB}}^{n}}$$

Assuming uniform and linear distribution of hydrostatic pressure along direction AB on the shear plane, the normal stress *F*_sn_ on the shear plane was expressed as:

(11) $$F_{\mathrm{sn}}=\frac{P_{A}+P_{B}}{2}l_{\mathrm{AB}}w$$

According to the geometric relationship in Figure 5, the angle *θ* between the chip formation force *F*_r_ and the shear force *F*_s_ can be determined, as expressed in Equation (12):

(12) $$\tan\theta =\frac{F_{\mathrm{sn}}}{F_{s}}=1+2(\frac{\pi }{4}-\phi )-\frac{C_{0}}{\lambda }n_{\mathrm{eq}}$$

The primary shear plane AB, where the maximum shear strain rate is located, divided the shear zone into two unequal regions. Along this zone, from AB to the initial shear line CD, the thickness is *λ* times the entire shear band thickness. According to the segmented power-law distribution hypothesis of shear strain rates proposed by Li [29], the strain-rate field in the shear zone can be described as follows:

(13) $$\dot{\gamma }=\left\{ \begin{array}{l} \frac{{\dot{\gamma }}_{\mathrm{AB}}}{{\left(\lambda h\right)}^{q}}y^{q}\;\;\;\;\;\;\;\;\;\;\;\;\;\;\;\;\;\;\;\;\;\;y\in \left[0,\lambda h\right]\\ \frac{{\dot{\gamma }}_{\mathrm{AB}}}{{\left(1-\lambda \right)}^{q}h^{q}}{\left(h-y\right)}^{q}\;\;\;y\in \left[\lambda h,h\right] \end{array}\right.$$

where ${\dot{\gamma }}_{AB}$ represents the maximum shear strain rate, *λ* is the coefficient of inequality, and *h* denotes the thickness of the shear zone. The coefficient (*q*) is an indeterminate parameter used to characterize the non-uniform distribution of tangential velocity in the primary deformation zone. For low-speed cutting, the value of *q* was 3 as reported by Li [29].

The strain at the primary shear plane ($\gamma_{\mathrm{AB}}$), the maximum shear strain rate (${\dot{\gamma }}_{\mathrm{AB}}$) and the non-uniform division coefficient (*λ*) were calculated by Equation (14):

(14) $$\left\{ \begin{array}{l} \gamma_{\mathrm{AB}}=\frac{\lambda \cos\gamma_{0}}{\sin\phi \cos(\phi -\gamma_{0})}\\ {\dot{\gamma }}_{\mathrm{AB}}=\frac{(q+1)v\cos\gamma_{0}}{h\cos(\phi -\gamma_{0})}\\ \lambda =\frac{\cos\phi \cos(\phi -\gamma_{0})}{\cos\gamma_{0}} \end{array}\right.$$

The values of equivalent plastic strain ($\varepsilon_{\mathrm{AB}})$ and equivalent plastic strain rate (${\dot{\varepsilon }}_{\mathrm{AB}}$) were calculated by Equation (15) based on the von Mises criterion.

(15) $$\left\{ \begin{array}{l} \varepsilon_{\mathrm{AB}}=\frac{\gamma_{\mathrm{int}}}{\sqrt{3}}\\ {\dot{\varepsilon }}_{\mathrm{AB}}=\frac{{\dot{\gamma }}_{\mathrm{int}}}{\sqrt{3}} \end{array}\right.$$

In high-speed cutting of high-temperature alloys, thermal conduction in the shear zone can be neglected, hence the boundaries of the shear band can be considered adiabatic. The temperature *T*_AB_ at the shear band AB was calculated by Equation (16):

(16) $$T_{\mathrm{AB}}=T_{r}+\eta \Delta T_{\mathrm{AB}}$$

(17) $$\Delta T_{\mathrm{AB}}=\frac{(1-\kappa )F_{s}\cos a}{\rho Sa_{1}b\cos(\phi -a)}$$

where Δ*T*_AB_ represents the temperature rise in the primary shear zone of the material due to plastic strain, *T*_r_ is the initial temperature of the workpiece (taken as 25 °C), *η* is the proportion of total shear energy converted into heat (assumed as 1 in this paper), *S* is the specific heat, and *ρ* is the density of the workpiece.

*κ* represents the thermal distribution coefficient of the shear zone, which was calculated by Equation (18):

(18) $$\kappa =\left\{ \begin{array}{l} 0.5-0.35\mathrm{lg}(R_{T}\tan\phi )\;\;\;\;0.04\leq R_{T}\tan\phi \leq 10.0\\ 0.3-0.15\mathrm{lg}(R_{T}\tan\phi )\;\;\;\;R_{T}\tan\phi \geq 10.0 \end{array}\right.$$

where *R*_T_ is an intermediate parameter. According to the empirical formula proposed by Boothroyd [30], its expression was as shown in Equation (1):

(19) $$R_{T}=\frac{S\rho va_{p}}{K}$$

where *K* represents the thermal conductivity coefficient of the workpiece.

The expression for the normal pressure ($\sigma_{N}^{\prime }$) at point A near the cutting-edge position can be obtained by combining the average shear flow and stress on the shear plane.

According to the average shear flow stress on the shear plane, the expression for the normal pressure ($\sigma_{N}^{\prime }$) at point A near the cutting edge was derived as Equation (20):

(20) $$\sigma_{N}^{\prime }=\tau_{\mathrm{AB}}\left(1+\frac{\pi }{2}-2\gamma_{0}-2C_{0}n_{\mathrm{eq}}\right)$$

Assuming the second shear zone is rectangular with uniformly distributed normal stress on the tool rake face, the corrected length of the chip–tool contact (*H*) was calculated by Equation (21):

(21) $$H=\frac{a_{1}\sin\theta }{\cos\beta \sin\phi }\left[1+\frac{(q+1)C_{0}n_{\mathrm{eq}}}{3[2\lambda +4\lambda \left(\frac{\pi }{4}-\phi \right)-(q+1)C_{0}n_{\mathrm{eq}}}\right]$$

Assuming that the flow stress in the chip is uniformly distributed along the tool–chip contact surface, the equations for the shear stress (*τ*_int_) along the tool–chip separation surface and the stress (*σ*_N_) at point A can be expressed as follows:

(22) $$\left\{ \begin{array}{l} \tau_{\mathrm{int}}=\frac{F_{f}}{Hw}\\ \sigma_{N}=\frac{F_{\mathrm{ns}}}{Hw} \end{array}\right.$$

According to the von Mises criterion, the equivalent strain and strain rate on the tool–chip contact surface can be expressed as follows:

(23) $$\left\{ \begin{array}{l} \varepsilon_{\mathrm{int}}=\frac{\gamma_{\mathrm{int}}}{\sqrt{3}}=\frac{1}{\sqrt{3}}(2\gamma_{\mathrm{AB}}+\gamma_{M})\\ {\dot{\varepsilon }}_{\mathrm{int}}=\frac{{\dot{\gamma }}_{\mathrm{int}}}{\sqrt{3}}=\frac{1}{\sqrt{3}}\frac{v_{c}}{\delta a_{2}} \end{array}\right.$$

where *δ* represents the ratio of the thickness of the second deformation zone (Δ*S*_2_) to the chip thickness (*a*_chip_). The value of *δ* ranges from 0.005 to 0.2, and its final value was determined through an iterative enumeration process.

The average temperature at the tool–chip contact interface can be expressed as:

(24) $$\left\{ \begin{array}{l} T_{\mathrm{int}}=T_{W}+\eta \left[\left(\frac{1-\kappa }{\rho Sa_{p}w}\right)\left(\frac{F_{s}\cos\gamma_{0}}{\cos(\phi -\gamma_{0})}\right)\right]+\psi \Delta T_{c}10^{\left[0.06-0.195\delta {\left(\frac{R_{T}a_{\mathrm{chip}}}{H}\right)}^{0.5}+0.5\mathrm{lg}\left(\frac{R_{T}a_{\mathrm{chip}}}{H}\right)\right]}\\ \Delta T_{c}=\frac{F_{\mathrm{fc}}\sin\phi }{\rho Sa_{p}w\cos(\phi -\gamma_{0})} \end{array}\right.$$

where the correction factor (*Ψ*) represents the ratio of the temperature rise at the tool–chip contact area to the maximum temperature rise within the chip. In this paper, its value was set to 0.9 [29]. Δ*T*_c_ denotes the average temperature rise within the chip.

The flowing stress on the tool–chip contact interface $\left(\tau_{\mathrm{chip}}\right)$ was calculated by Equation (25) based on the above information [31].

(25) $$\tau_{chip}=\frac{1}{\sqrt{3}}\left[A+B{\varepsilon_{\mathrm{int}}}^{n}\right]\left[1+C\ln\left(\frac{{\dot{\varepsilon }}_{\mathrm{int}}}{{\dot{\varepsilon }}_{0}}\right)\right]\left[1-{\left(\frac{T_{\mathrm{int}}-T_{r}}{T_{w}-T_{r}}\right)}^{m}\right]$$

**Figure 6:**
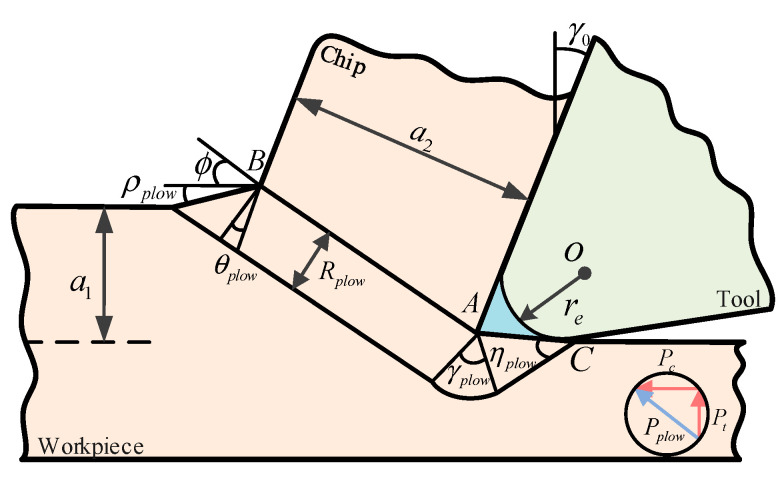
Schematic diagram of plow force model.

The unequal division model was based on the assumption that the tool tip was perfectly sharp, ignoring the plowing effect of the tool’s blunt edge. However, during metal turning processes, when the feed force is small, the influence of plowing forces can be further amplified [32]. In order to ensure the accuracy of the J–C constitutive model, the effect of the third deformation zone during the turning process cannot be ignored. Therefore, the slip line field model proposed by Waldorf et al. [33] was adopted to analyze the calculation model of plowing forces in the cutting process, as shown in Figure 6.

The sector angles *θ*_plow_ and *γ*_plow_ were calculated by Equation (26) based on the geometric relationships in Figure 6.

(26) $$\left\{ \begin{array}{l} \theta_{\mathrm{plow}}=\frac{\pi }{4}-\rho_{\mathrm{plow}}-\phi \\ \gamma_{\mathrm{plow}}=\eta_{\mathrm{plow}}+\phi -\arcsin(\sqrt{2}\sin p_{\mathrm{plow}}\sin\eta_{\mathrm{plow}}) \end{array}\right.$$

where *ρ*_plow_ represents the angle between the protrusion caused by the blunt radius *r*_e_ of the tool edge and the machined surface. When the radius of the tool tip is small, *ρ*_plow_ can be taken as 10°. In this paper, considering a larger radius of the tool tip, *ρ*_plow_ was set to 0°.

The angle *η*_plow_ between the slip line and AC was calculated by Equation (27) based on the slip line field theory.

(27) $$\eta_{plow}=0.5\;\arccos\;\mu_{plow}$$

where *μ*_plow_ represents the friction factor of the metal dead zone, where the flow stress of the chip in the contact area of the chip curl is approximately equal to the shear stress. *μ*_plow_ was taken as 0.99. *R*_plow_ denotes the radius of the sector region.

(28) $$R_{\mathrm{plow}}=\sin\eta_{\mathrm{plow}}\sqrt{{\left[r_{e}\tan\left(\frac{\pi }{4}+\frac{\gamma_{0}}{2}\right)+\frac{\sqrt{2}R_{\mathrm{plow}}\sin p_{\mathrm{plow}}}{\tan\left(\frac{\pi }{4}+\frac{\gamma_{0}}{2}\right)}\right]}^{2}+2{\left[R_{\mathrm{plow}}\sin p_{\mathrm{plow}}\right]}^{2}}$$

The plowing forces *P*_c_ along the cutting direction and *P*_t_ perpendicular to the cutting direction were calculated by Equation (29):

(29) $$\left\{ \begin{array}{l} P_{c}=\frac{\tau_{\mathrm{AB}}R_{\mathrm{plow}}w}{\sin\eta_{\mathrm{plow}}}\left[\begin{array}{l} (1+2\theta_{\mathrm{plow}}+2\gamma_{\mathrm{plow}}+\sin(2\eta_{\mathrm{plow}}))\sin(\phi -\gamma_{\mathrm{plow}}+\eta_{\mathrm{plow}})+\\ \cos(2\eta_{\mathrm{plow}})\cos(\phi -\gamma_{\mathrm{plow}}+\eta_{\mathrm{plow}}) \end{array}\right]\\ P_{t}=\frac{\tau_{\mathrm{AB}}R_{\mathrm{plow}}w}{\sin\eta_{\mathrm{plow}}}\left[\begin{array}{l} (1+2\theta_{\mathrm{plow}}+2\gamma_{\mathrm{plow}}+\sin(2\eta_{\mathrm{plow}}))\cos(\phi -\gamma_{\mathrm{plow}}+\eta_{\mathrm{plow}})-\\ \cos(2\eta_{\mathrm{plow}})\sin(\phi -\gamma_{\mathrm{plow}}+\eta_{\mathrm{plow}}) \end{array}\right] \end{array}\right.$$

The total cutting force is the sum of the plowing force and the cutting forming force, which was expressed by Equation (30):

(30) $$\left\{ \begin{array}{l} F_{C}=F_{c}+P_{c}\\ F_{T}=F_{t}+P_{t} \end{array}\right.$$

## 4. Inverse Identification of the J–C Constitutive Model

### 4.1. The Inverse Identification Process for the Constitutive Model

The inverse identification of the J–C constitutive model for materials is based on experimental data and the orthogonal cutting theory model to determine unknown parameters within the constitutive model. These parameters are used to describe the deformation characteristics of the material under conditions of high strain rates and temperatures. The inversion process typically involves optimization and inverse problem-solving methods to identify the most realistic parameter values, ensuring accurate predictions of the material’s mechanical response under complex loading conditions.

Recently, optimization algorithms used for constitutive parameter identification have commonly included genetic algorithms (GA) and particle swarm optimization (PSO) [18]. In order to improve upon the tendency of traditional algorithms to converge to local optima, this paper employed a genetic–particle swarm algorithm (GA–PSO). The GA–PSO combines the global search capabilities of GA with the local search abilities of PSO, creating a dynamic optimization mechanism. During the initialization phase, the algorithm generates a population of multiple individuals and sets the initial positions and velocities for the particle swarm. Subsequently, genetic operations such as selection, crossover, and mutation are applied to the population to maintain diversity and adapt to the complex search space. On this basis, particles update their velocities and positions based on their own best experiences and those of their neighbors, enabling effective exploration of the solution space. By integrating the strengths of both algorithms, GA–PSO can quickly converge to potential optimal solutions while avoiding local optima. The main advantages of GA–PSO lie in its powerful global search capability and rapid convergence speed. By combining the diversity maintenance mechanisms of GA with the collaborative search strategies of PSO, GA–PSO effectively addresses nonlinear, non-convex, and high-dimensional problems. Compared to single algorithms, GA–PSO offers greater adaptability and flexibility in handling complex optimization tasks [34].

In the absence of relevant literature on the J–C constitutive equation for GH4198 high-temperature alloy, approximate ranges, established based on the equation for GH4169 high-temperature alloy [17], were set as follows: 0 ≤ *C* ≤ 0.1, 1 ≤ *m* ≤ 2. The fitness function, which determines the convergence speed of the algorithm and influences the feasibility of solutions, must satisfy conditions such as being single-valued, consistent, and continuous. The fitness value was determined by minimizing the average error between the predicted stress (${\bar{\tau }}_{AB}$) and shear stress (${\bar{\tau }}_{\exp}$) obtained from orthogonal cutting experiments, as shown in Equation (31).

(31) $$\left\{ \begin{array}{l} f(c,m)=\min\frac{1}{N}\sum_{i=1}^{N}{\left(\frac{{\bar{\tau }}_{\mathrm{AB}}(i)}{{\bar{\tau }}_{\exp}(i)}-1\right)}^{2}\\ \mathrm{subject}\;\mathrm{to}:\left\{ \begin{array}{l} C_{\min}\leq C\leq C_{\max}\\ m_{\min}\leq m\leq m_{\max} \end{array}\right. \end{array}\right.$$

where *N* represents the total number of cutting tests conducted, and *i* denotes the test number.

**Figure 7:**
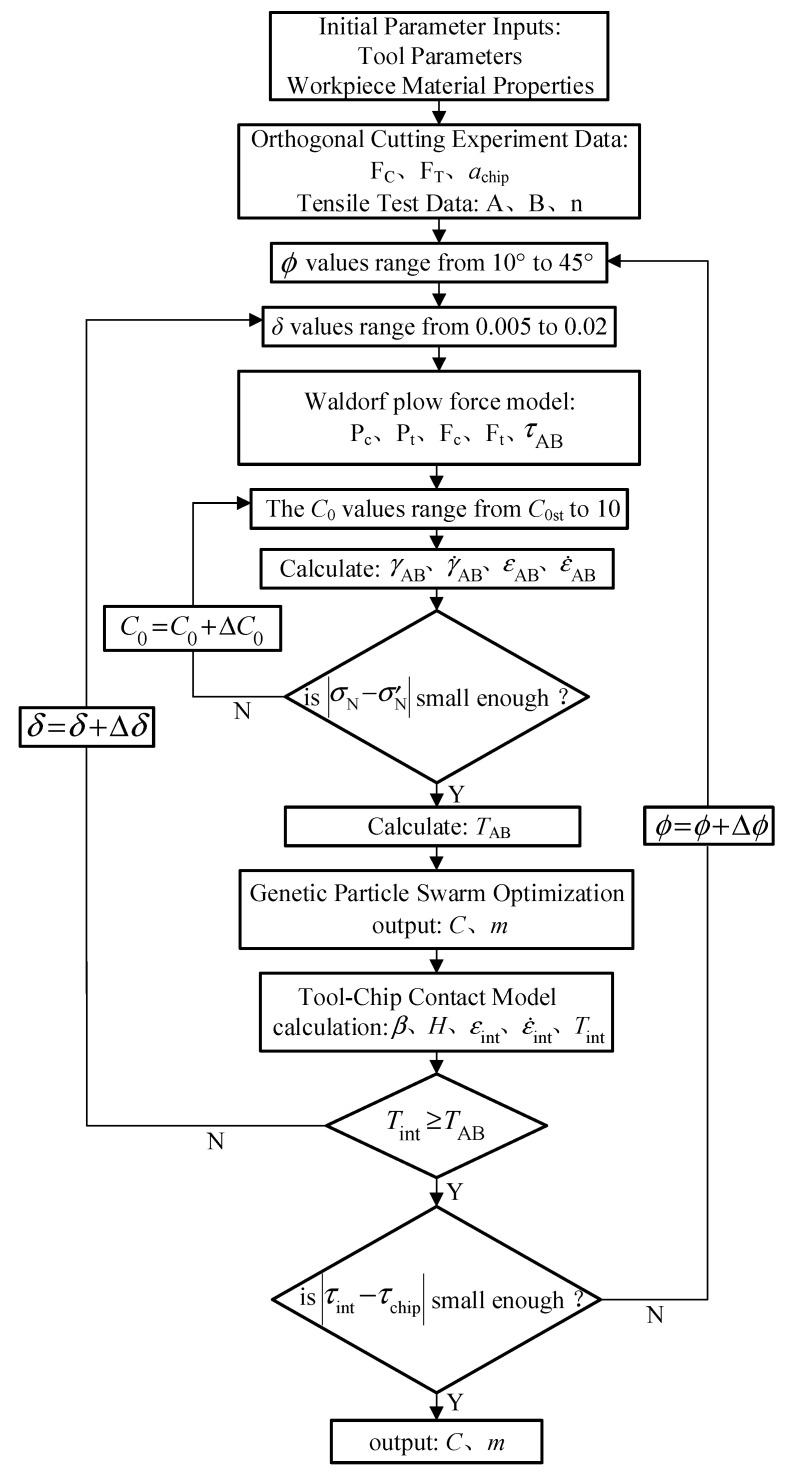
Flowchart of constitutive parameter inversion.

Settings of relevant parameters in the genetic–particle swarm algorithm: the learning factor was set to 1.49445, with maximum (*W*_max_) and minimum (*W*_min_) inertia factors of 0.9 and 0.4 respectively. The crossover probability (*P*_c_) was 0.5, mutation probability (*P*_m_) was 0.05, population size was 20, and the number of iterations was 150. During the algorithm compilation process, initial calculations based on experimental data *F*_C_, *F*_T_, *a*_2_, *A*, *B*, and *n*, combined with the Waldorf plowing model, determined $\varepsilon_{\mathrm{AB}}$, ${\dot{\varepsilon }}_{\mathrm{AB}}$, and *T*_AB_ in the primary shear zone. Subsequently, the values of *C* and *m* obtained from the genetic–particle swarm algorithm were substituted into the decision criteria to produce the final values. The specific flowchart of the J–C constitutive model inversion algorithm is illustrated in Figure 7.

**Figure 8:**
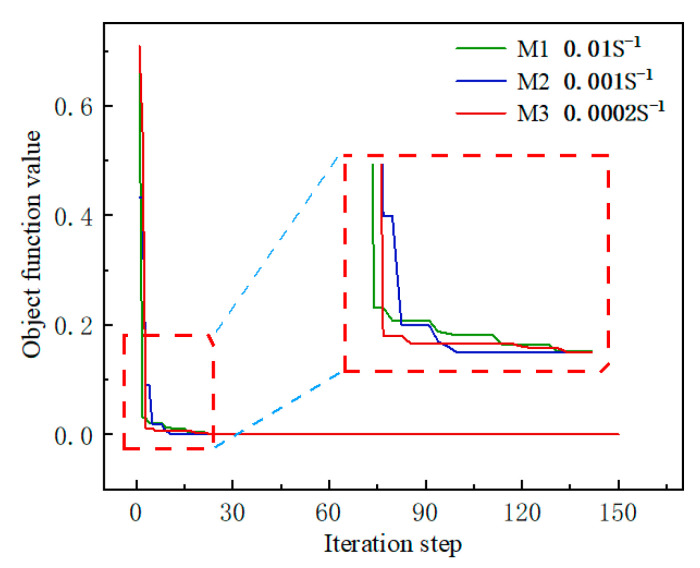
The trend of fitness values with the number of iterations.

The optimal fitness of the J–C constitutive parameters decreased gradually with the iteration process when running the inversion program in MATLAB, stabilizing within 30 iterations. To prevent the algorithm from converging to local minima, the program was executed multiple times, ensuring improved reliability of the inversion results for the parameters *C* and *m*. The iterative convergence process for three different reference strain rates is shown in Figure 8.

### 4.2. The Results and Analysis of the GH4198 Constitutive Model

The shear angle ϕ is the angle between the shear slip plane and the cutting velocity *v*. It is closely related to factors such as friction angle, cutting forces, surface roughness, tool wear, etc., during the cutting process, and is one of the key parameters used to characterize the extent of chip deformation.

(32) $$\phi =\arctan\frac{\cos\gamma_{0}}{a_{\mathrm{chip}}/a_{p}-\sin\gamma_{0}}$$

The chip thickness (*a*_chip_) was measured to calculate the shear angle ϕ corresponding to different machining parameters based on the orthogonal cutting experiment. These values were then compared and analyzed against the predicted shear angle ϕ_p_ from the orthogonal cutting model. The maximum prediction error for the shear angle was 8.8%, and the minimum was 3.2%, as shown in Table 4.

The parameters of the J–C constitutive model for GH4198 high-temperature alloy were inverted through the genetic–particle swarm algorithm. The inversion program was run 15 times at each reference strain rate. The average values of *C* are 0.063, 0.061, and 0.058, with standard deviations of 0.0088, 0.0042, and 0.0076, respectively. The average values of *m* are 1.753, 1.529, and 1.453, with standard deviations of 0.1467, 0.1245, and 0.0972, respectively. For instance, in the M2 group of experiments, the value of strain-rate sensitivity coefficient *C* was 0.059 at a reference strain rate of 0.001, and the thermal softening coefficient *m* was 1.586. It was observed that as the reference strain rate decreased, the strain-rate sensitivity coefficient remained essentially unchanged while the thermal softening coefficient decreased, as shown in Table 5.

We combined the initial yield strength, strain hardening coefficient, and processing hardening exponent obtained from the quasi-static tensile tests at different reference strain rates as mentioned above, as shown in Table 3. Finally, the J–C constitutive equation for GH4198 high-temperature alloy was derived, as shown in Equation (33).

(33) $$\left\{ \begin{array}{l} M1:\;\tau_{\mathrm{AB}}=\frac{1}{\sqrt{3}}\left[1136.234+1179.481\varepsilon_{\mathrm{AB}}^{0.502}\right]\left[1+0.060\ln\left(\frac{{\dot{\varepsilon }}_{\mathrm{AB}}}{{\dot{\varepsilon }}_{0}}\right)\right]\left[1-{\left(\frac{T_{\mathrm{AB}}-T_{r}}{T_{W}-T_{r}}\right)}^{1.772}\right]\\ M2:\;\tau_{\mathrm{AB}}=\frac{1}{\sqrt{3}}\left[1138.895+1156.603\varepsilon_{\mathrm{AB}}^{0.541}\right]\left[1+0.059\ln\left(\frac{{\dot{\varepsilon }}_{\mathrm{AB}}}{{\dot{\varepsilon }}_{0}}\right)\right]\left[1-{\left(\frac{T_{\mathrm{AB}}-T_{r}}{T_{W}-T_{r}}\right)}^{1.586}\right]\\ M3:\;\tau_{\mathrm{AB}}=\frac{1}{\sqrt{3}}\left[1157.537+1138.349\varepsilon_{\mathrm{AB}}^{0.686}\right]\left[1+0.060\ln\left(\frac{{\dot{\varepsilon }}_{\mathrm{AB}}}{{\dot{\varepsilon }}_{0}}\right)\right]\left[1-{\left(\frac{T_{\mathrm{AB}}-T_{r}}{T_{W}-T_{r}}\right)}^{1.354}\right] \end{array}\right.$$

## 5. Validation of Constitutive Model Based on Finite-Element Analysis

In the cutting process, the stress, strain, and temperature distributions in the primary shear zone are non-uniform, and the frictional interaction between the tool and chip is complex, making precise prediction challenging for theoretical models. Finite-element simulation and numerical analysis complement theoretical models effectively. ABAQUS (Explicit) conducts dynamic analysis by explicit time integration of the dynamic finite-element equations, suitable for analyzing transient, nonlinear, large deformation problems [35,36,37].

### 5.1. Establishment of the Finite-Element Model

**Figure 9:**
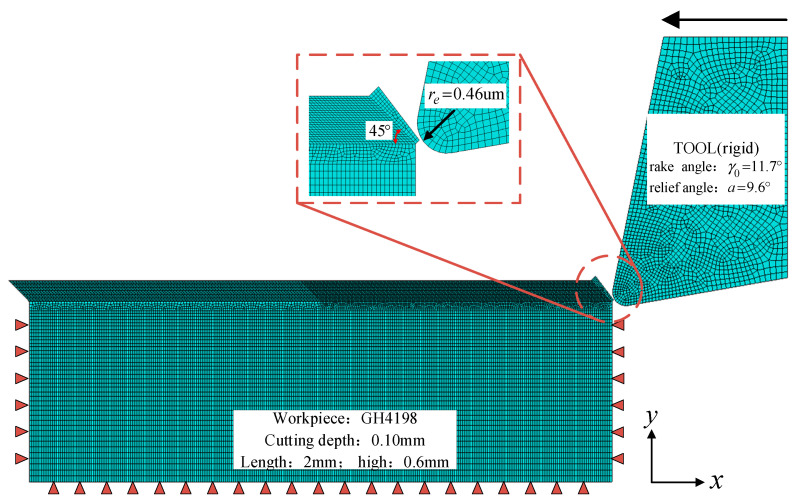
Orthogonal cutting finite-element model.

In order to validate the effectiveness of the J–C constitutive equation parameters, a thermal–mechanical coupled model of two-dimensional cutting was established using ABAQUS (2022), as shown in Figure 9. In developing the model, the impact of mesh size on the simulation results was taken into account, and mesh optimization was performed with a mesh size of 0.05 mm. The mesh employed four-node plane strain bilinear thermal–mechanical coupled reduced integration elements [38]. Fixed constraints were applied on the workpiece to restrict its motion, and relative motion between the tool and workpiece was achieved by applying loads on the tool. Tool wear was not considered, and the geometric model of the tool was treated as rigid. To prevent excessive mesh distortion during tool entry into the workpiece, the mesh of the cutting layer was tilted relative to the plane by 45° [39].

During orthogonal cutting processes, friction between the tool and workpiece generated significant heat, causing an increase in chip temperature and resulting in thermal softening of the material. Therefore, in the two-dimensional finite-element simulation of orthogonal cutting, consideration was needed for how the material properties of the workpiece varied with temperature. The physical properties of GH4198 high-temperature alloy material changed with temperature, as shown in Table 6.

The criteria for material separation influenced the chip morphology and the separation position of the chip from the machined surface [40]. In order to establish a model for the separation of the chip from the workpiece matrix, The Johnson–Cook (J–C) damage model was typically used as the damage criterion. The J–C damage model was based on the equivalent plastic strain at the integration point of the element. When the damage factor *ω* of a mesh element reached a designated value, the element then was deleted [41].

(34) $$\omega =\sum \frac{\Delta \bar{\varepsilon }}{{\bar{\varepsilon }}_{\mathrm{JC}}}$$

where *ω* is the damage state variable, $\Delta \bar{\varepsilon }$ is the equivalent plastic strain increment, and ${\bar{\varepsilon }}_{\mathrm{JC}}$ is the initial damage strain determined by the J–C model. The equation for J–C fracture strain was calculated by Equation (35).

(35) $${\bar{\varepsilon }}_{f}=\left[D_{1}+D_{2}\exp\left(D_{3}\frac{P}{\bar{\sigma }}\right)\right]\left[1+D_{4}\ln\frac{{\dot{\varepsilon }}_{\mathrm{AB}}}{{\dot{\varepsilon }}_{0}}\right]\left[1+D_{5}\frac{T_{\mathrm{AB}}-T_{r}}{T_{w}-T_{r}}\right]$$

where *P* represents static hydrostatic pressure, and *D*_1_, *D*_2_, *D*_3_, *D*_4_, and *D*_5_ are material failure parameters, as shown in Table 7. This study conducted quasi-static tensile experiments on notched round bars at room temperature, smooth round bar tensile experiments at various strain rates at room temperature, and quasi-static tensile simulation experiments on smooth round bars at different temperatures. Stress–strain curves for each set were plotted, and data for D1–D5 were obtained.

The local friction between the tool and the chip was discontinuous and correlated with the distribution of normal stress and frictional stress [42]. A friction model consisting of adhesive and sliding friction regions was employed.

(36) $$\left\{ \begin{array}{l} \tau =\tau_{p}\;\;\;\;u\sigma_{n}<\tau_{p}\;\\ \tau =u\sigma_{n}\;\;\;u\sigma_{n}\geq \tau_{p} \end{array}\right.$$

where *τ* represents the friction stress, $\sigma_{n}$ denotes the normal stress between two contacting surfaces, *u* is the coefficient of friction in the sliding region, and $\tau_{p}$ stands for the critical shear yield stress.

### 5.2. Constitutive Model Validation and Discussion

**Figure 10:**
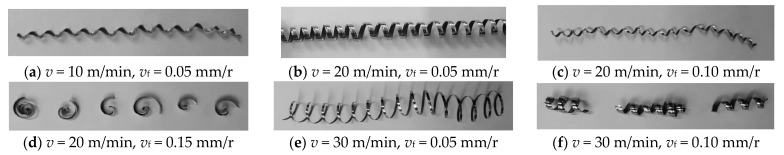
Comparison of chip morphology.

When the cutting speed *v* was 20 m/min, with increasing feed rate, the cutting resistance increased, as shown in Figure 10. Internal thermal stresses within the chips gradually increased, leading to the generation of numerous tiny cracks in the deformation zone of the chips. These cracks then expanded, causing chip fracture, transitioning the chips from coiled chips to fragmented chips. When the feed rate *v*_f_ was 0.05 mm/r, with increasing cutting speed, the temperature in the tool–chip contact area increased. This enhanced the thermal softening effect of the workpiece material, resulting in an increased curling radius of the chips.

The cutting parameters corresponding to NO. i (i = 1, 2, 5, 6, 8) in Table 8 are as shown in Table 4, with the reference strain rate ${\dot{\varepsilon }}_{0}$ being 0.001. The results of finite-element simulation of chip characteristics were compared with experimental values. The maximum predicted error in chip thickness was 12.5%, with the minimum being 0.7%; the maximum predicted error in serration depth was 10.7%, with the minimum being 3.9%; the maximum predicted error in cutting force was 24.5%, with the minimum being 15.2%. Additionally, the simulated macroscopic morphology of the chips correlated well with the experimental results shown in Figure 10.

### 5.3. The Influence of Constitutive Parameters on Simulated Observations

The J–C constitutive model had a significant impact on the formation of sawtooth-shaped chips in finite-element simulations of cutting [43]. In order to analyze the influence of strain-rate sensitivity coefficient *C* and thermal softening exponent *m* on simulated observables, an orthogonal experiment was designed with six levels for each constant, varying within ±100%. The variations of the strain-rate sensitivity coefficient *C* and thermal softening exponent *m* are shown in Table 9.

**Figure 11:**
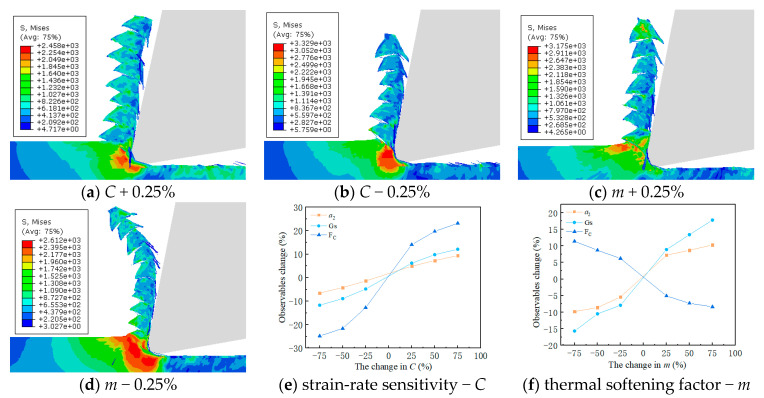
The influence of changes in constitutive parameters on cutting simulation.

The effects of changes in the strain-rate sensitivity coefficient *C* and thermal softening exponent *m* on simulated observables at a reference strain rate ${\dot{\varepsilon }}_{0}$ of 0.001, cutting speed *v* of 20 m/min, and feed rate *v*_f_ of 0.1 mm/r, are shown in Figure 11. As the strain-rate sensitivity coefficient *C* and thermal softening exponent *m* increased, both chip thickness and serration depth showed an increasing trend. Increasing the strain-rate sensitivity coefficient *C* and decreasing thermal softening exponent *m* resulted in an increasing trend in cutting force.

## 6. Conclusions

In order to obtain the constitutive model of the new forged and cast alloy GH4198 material, this paper considered the influence of tool nose radius on the cutting process and proposed a method for inverse identification of the Johnson–Cook (J–C) constitutive model based on a genetic–particle swarm algorithm. An orthogonal cutting thermo-mechanical coupled simulation model was established to validate the effectiveness of the J–C constitutive model. Furthermore, the impact of the J–C constitutive model on simulated observables was analyzed. The main conclusions drawn from this study are summarized as follows:

1. Quasi-static tensile tests were conducted at different strain rates. The results indicated that at lower strain rates, as the strain rate decreased, the material’s initial yield strength (*A*) and work hardening exponent (*n*) showed an increasing trend, while the strain hardening coefficient (*B*) showed a decreasing trend.
2. Orthogonal cutting experiments were conducted, and a genetic–particle swarm algorithm model based on the unequal division shear theory was proposed. The results indicated that the maximum predicted error of shear angle was 8.8%, validating that the method of determining shear angle through exhaustive iteration was reasonable.
3. The cutting finite-element model was established based on the inverse identification of the J–C constitutive model. The simulation results were compared with experimental values, showing high consistency, which demonstrated the effectiveness of the J–C constitutive model.
4. The sensitivity analysis of the J–C constitutive model on simulated observables was conducted. The results revealed that the thermal softening coefficient (*m*) had a greater influence on the simulation results of chip geometry features, while the strain-rate hardening coefficient (*C*) had a more significant effect on the simulation results of cutting forces.

The methods proposed in this paper predicted the constitutive model of GH498 high-temperature alloy effectively. Future research will focus on developing a slip line field model based on unequal division shear theory to better fit the formation of serrated chips, thereby further enhancing the predictive accuracy of the J–C constitutive model.

## Figures and Tables

**Table 1:** Chemical composition of the GH4198 high-temperature alloy.

Element	Cr	Co	W	Mo	Ti	Al	Nb	Ta	C	B	Zr	Ni
Percentage	13.0	20.5	2.3	3.8	3.8	3.4	1.0	2.5	0.05	0.015	0.05	Base

**Table 2:** Tensile test results of GH4198 high-temperature alloy.

Sample	${\dot{\varepsilon }}_{0}$	Max Load (N)	Max Displacement (mm)	Tensile Strength (MPa)
M1	0.01	28,569.2	4.653	1454.232
M2	0.001	30,378.3	4.635	1547.154
M3	0.0002	29,971.0	5.033	1526.412

**Table 3:** Fitting results of quasi-static tensile test.

Sample	${\dot{\varepsilon }}_{0}$	*A* (Mpa)	*B* (Mpa)	*n*
M1	0.01	1136.234	1179.481	0.502
M2	0.001	1138.895	1156.603	0.541
M3	0.0002	1157.537	1138.349	0.686

**Table 4:** Orthogonal cutting parameters and measurement results of GH4198 high-temperature alloy.

NO.	Feed Rate *v*_f_ (mm/r)	Cutting Speed *v* (m/min)	Chip Thickness *a*_chip_ (mm)	*F*_C_ (N)	*F*_T_ (N)	Experiment ϕ (°)	Prediction ϕ_p_ (°)	Error
1	0.05	10	0.186	654.3	502.8	16.0	17.4	8.8%
2	0.05	20	0.133	613.6	481.3	21.7	22.4	3.2%
3	0.05	30	0.098	533.8	464.4	31.4	29.1	7.3%
4	0.10	10	0.287	967.4	815.7	20.2	21.3	5.5%
5	0.10	20	0.229	906.3	763.9	25.2	24.4	3.2%
6	0.10	30	0.160	853.6	682.5	35.1	37.0	5.4%
7	0.15	10	0.351	1107.5	916.3	23.9	25.4	6.3%
8	0.15	20	0.310	1030.7	883.6	27.8	29.5	6.1%
9	0.15	30	0.281	974.5	784.2	37.6	34.5	8.2%

**Table 5:** J–C constitutive model parameters of GH4198.

Sample	${\dot{\varepsilon }}_{0}$	*C*	*m*
M1	0.01	0.060	1.772
M2	0.001	0.059	1.586
M3	0.0002	0.060	1.354

**Table 6:** GH4198 high-temperature alloy material performance parameters [4].

NO.	Temperature (°)	Density (g·cm^−3^)	Poisson’s Ratio	Young’s Modulus (GPa)	Specific Heat (J·g^−1^·K^−1^)	Expansion Coefficient (10^−6^ °C^−1^)	Thermal Conductivity (W·m^−1^·K^−1^)
1	25	8.30	0.28	229	1.08	11.69	23.2
2	100		0.29	226	0.95	13.19	22.8
3	200		0.29	220	1.05	13.84	27.7
4	300		0.30	213	1.08	14.05	30.6
5	400		0.30	207	1.19	14.03	36.4
6	500		0.30	200	1.16	16.51	37.4
7	600		0.31	193	1.08	16.65	36.9
8	700		0.31	185	0.86	20.37	31.1
9	800		0.32	176	0.74	21.53	27.6
10	900		0.32	166	0.56	22.69	21.2

**Table 7:** The material failure parameters of GH4198 high-temperature alloy.

*D*_1_	*D*_2_	*D*_3_	*D*_4_	*D*_5_
0.034	0.015	1.357	−0.773	1.906

**Table 8:** Validation of experimental and simulation results.

NO.	Experimental	Simulation	Simulation Observables	R_exp_	R_sim_	Error
1	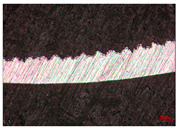	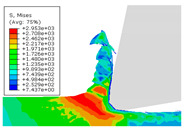	*a*_chip_	0.181	0.167	7.7%
			Gs	0.103	0.114	10.7%
			F_C_	654.3	511.7	21.8%
2	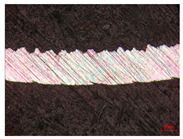	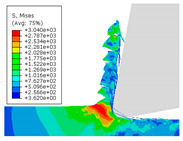	*a*_chip_	0.133	0.127	4.5%
			Gs	0.122	0.133	9.0%
			F_C_	613.6	463.2	24.5%
5	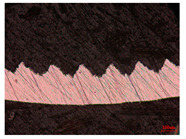	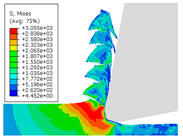	*a*_chip_	0.229	0.221	3.5%
			Gs	0.357	0.384	7.6%
			F_C_	906.3	714.6	21.2%
6	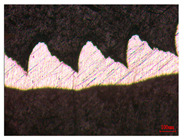	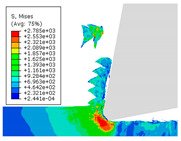	*a*_chip_	0.160	0.140	12.5%
			Gs	0.584	0.607	3.9%
			F_C_	853.6	667.8	21.7%
8	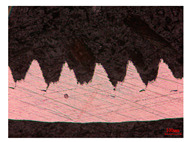	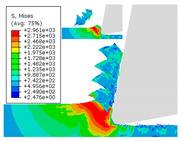	*a*_chip_	0.310	0.308	0.7%
			Gs	0.420	0.400	4.8%
			F_C_	1030.7	874.5	15.2%

**Table 9:** Changes in constitutive parameters at a reference strain rate of 0.001.

J–C	−75%	−50%	−25%	+0%	+25%	+50%	+75%
*C*	0.015	0.030	0.044	0.059	0.074	0.089	0.103
*m*	0.397	0.793	1.190	1.586	1.983	2.379	2.776

## Data Availability

The original contributions presented in the study are included in the article, further inquiries can be directed to the corresponding author.
